# Advances in the implications of the gut microbiota on the treatment efficacy of disease-modifying anti-rheumatic drugs in rheumatoid arthritis

**DOI:** 10.3389/fimmu.2023.1189036

**Published:** 2023-09-28

**Authors:** Junyu Fan, Ting Jiang, Dongyi He

**Affiliations:** ^1^ Department of Rheumatology, Guanghua Hospital Affiliated to Shanghai University of Traditional Chinese Medicine, Shanghai University of Traditional Chinese Medicine, Shanghai, China; ^2^ Department of Rheumatology, Shanghai Guanghua Hospital of Integrated Traditional Chinese and Western Medicine, Shanghai, China; ^3^ Institute of Arthritis Research in Integrative Medicine, Shanghai Academy of Traditional Chinese Medicine, Shanghai, China

**Keywords:** gut microbiota, dysbiosis, rheumatoid arthritis, immune response, DMARDs

## Abstract

Alterations in the composition or function of the gut microbiota are associated with the etiology of human diseases. Drug-microbiota interactions can affect drug bioavailability, effectiveness, and toxicity through various routes. For instance, the direct effect of microbial enzymes on drugs can either boost or diminish their efficacy. Thus, considering its wide range of metabolic capabilities, the gut microbiota is a promising target for pharmacological modulation. Furthermore, drugs can alter the microbiota and the mechanisms by which they interact with their host. Individual variances in microbial profiles can also contribute to the different host responses to various drugs. However, the influence of interactions between the gut microbiota and drugs on treatment efficacy remains poorly elucidated. In this review, we will discuss the impact of microbiota dysbiosis in the pathogenesis of rheumatoid arthritis (RA), and we will attempt to elucidate the crosstalk between the gut microbiota and disease-modifying anti-rheumatic drugs (DMARDs), with an emphasis on how drug-microbiota interactions affect the treatment efficacy in RA. We speculate that improved knowledge of these critical interactions will facilitate the development of novel therapeutic options that use microbial markers for predicting or optimizing treatment outcomes.

## 1 Introduction

Rheumatoid arthritis (RA) is one of the most common immune-mediated disorders. Its primary manifestation is inflammatory arthritis, characterized by symmetric, polyarticular pain and swelling, frequently affecting peripheral joints (1). Despite recent significant advances in RA therapy, overall remission remains unsatisfactory, and once bone or joint degradation has started it is virtually impossible to reverse (2). The poorly defined RA pathogenetic mechanisms may be related to the intricate relationship between genetic, environmental, and immunological responses, resulting in immune system dysregulation and lack of autoimmune tolerance.

Over the past years, a growing number of evidence from epidemiological and translational research suggests that interactions between mucosal sites and dysbiotic microbiota may play a causal role in RA development (3, 4). Notably, altered composition of microbial flora has been observed in the preclinical stages of RA patients. Furthermore, the gut contains more innate and adaptive immune cells than any other organ in the body (5, 6). The intricate linkages between altered gut microbiota and an immune system genetically predisposed to autoimmunity may be the basis of inflammatory arthritis. These changes in the gut microbiota may result from a systemic inflammation that affects the gut, or from an interplay between the environment and the innate immune system in individuals genetically prone to RA (7). Nevertheless, studies in early-stage RA patients and results from murine models indicate that these changes may predate the onset of arthritis, and constitute a veiled trigger of systemic inflammation (3).

The emergence of new innovative technologies has facilitated the investigation of the gut microbiota, which is pivotal in determining the clinical features and therapeutic responses of RA patients (8). Great advances have been made in relation to microbial diversity and the investigation of the bacterial metagenome in RA by sequencing analysis of the bacterial 16s ribosomal RNA (rRNA) and metagenomics (8, 9). In particular, a wide range of bacterial species associated with the RA clinical presentation spectrum has been identified in RA patients (10). Additionally, cohort studies have aided in establishing a link between gut microbiota and therapeutic response variability in RA (3, 10). Therefore, understanding the mechanisms of the effects disease-modifying anti-rheumatic drugs (DMARDs) exert on the gut microbiota, the consequences of gut dysbiosis in regulating treatment efficacy, and the approaches to restore microbial symbiosis in RA is crucial.

## 2 Microbiota and immune dysfunction in RA

**Figure 1:**
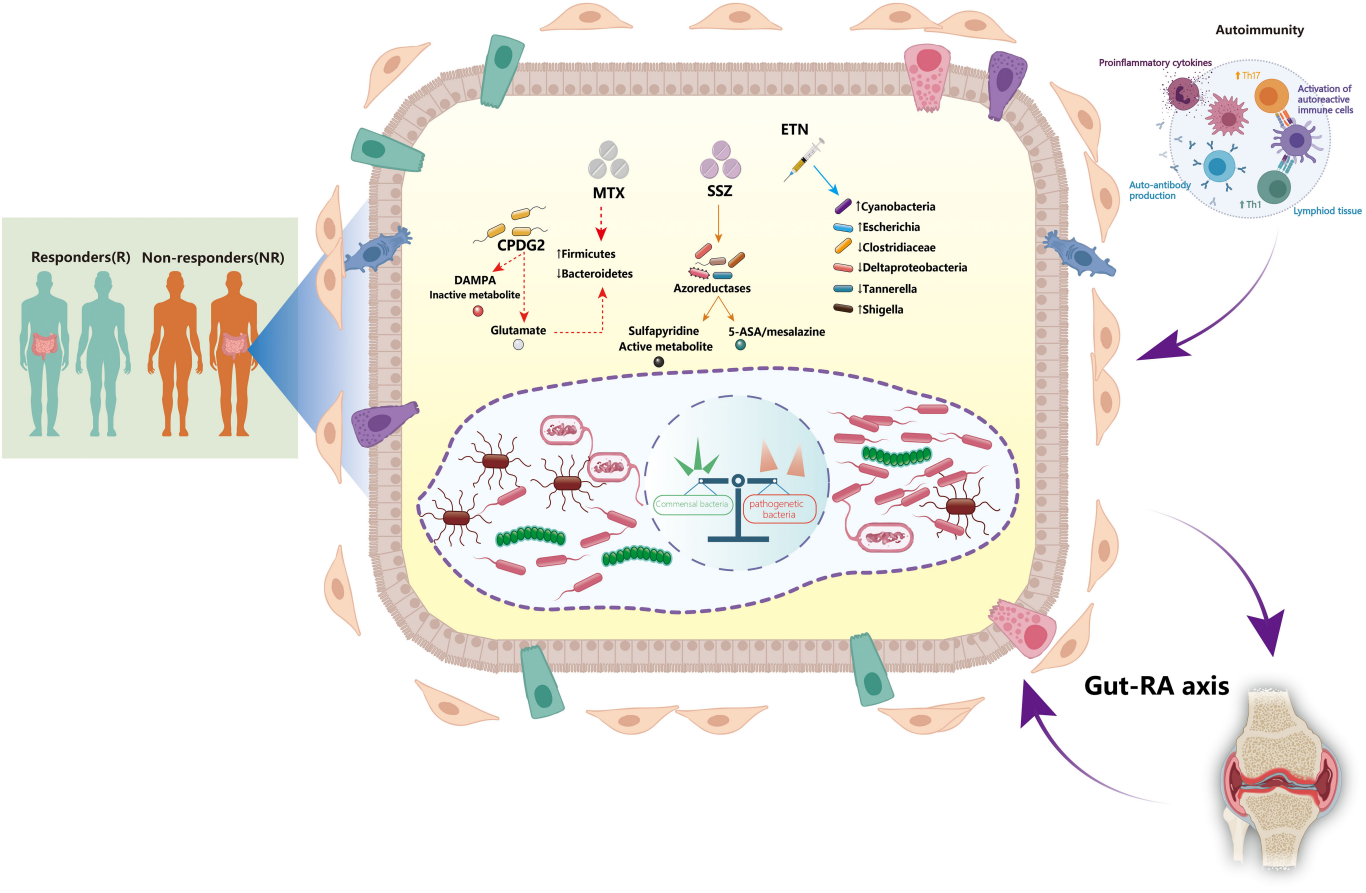
Interactions between the gut microbiota and disease-modifying anti-rheumatic drugs: implications for treatment efficacy in rheumatoid arthritis. The gut microbiota plays a critical role in the pathogenesis of rheumatoid arthritis (RA). The dynamic gut microbiota composition helps regulating host autoimmunity. Expansion of some opportunistic commensal bacteria may influence RA development by modifying the host’s microbiome, metabolic profile, and immune responses. The microbiota and its metabolite-associated signals are responsible for the activation and function of different immune cells. Autoreactive cells (e.g., Th1 and Th17 cells) are activated in lymphoid tissues, leading to inflammatory cytokine responses and production of autoantibodies. Certain disease-modifying anti-rheumatic drugs (DMARDs), such as methotrexate (MTX), sulfasalazine (SSZ), and etanercept (ETN), can directly affect the growth of gut microbiota. Furthermore, alteration of the gut microbiota may also contribute to the treatment efficacy of DMARDs by various mechanisms. CPDG2, carboxypeptidase-G2; DAMPA, 2,4-diamino-N(10)-methylpteroic acid; ETN, etanercept; MTX, methotrexate; SSZ, sulfasalazine; Th1, T helper 1 cells; Th17, T helper 17 cells.

Animal studies have demonstrated that alterations in the gut microbiota can affect local and systemic immunity, thereby resulting in joint inflammation (11, 12) (**Figure 1**). Antibiotics treatment has been shown to worsen arthritis in collagen-induced arthritis (CIA) mice, and to elevate levels of proinflammatory cytokines such as interleukin (IL)-6, interferon (IFN)-gamma, and IL-17 (13). *Desulfovibrio*, *Prevotella*, *Parabacteroides*, *Odoribacter*, *Acetatifactor*, *Blautia*, *Coprococcus*, and *Ruminococcus* genera were abundant, and levels of serum IL-17 and splenic CD4^+^ Th17 cells were elevated in arthritic mice, suggesting that the gut microbiota composition differs between CIA-susceptible and CIA-resistant mice (13). IL-17 and IL-1β production, and toll like receptor-2 (TLR2) and TLR4 activation, are increased in IL-1RA knockout mice, whereas these inflammatory responses are suppressed under germ-free conditions, attenuating arthritis development (14–16). Nevertheless, the clinical scores of these IL-RA knockout germ-free mice improved upon colonization with *Bifidobacterium bifidum* compared with those maintained under standard conditions (15). Maeda et al. discovered that transferring gut microbiota of RA patients to germ-free arthritis-prone SKG mice led to an increase in Th17 cells and severe arthritis. Further evidence revealed that co-culturing SKG dendritic cells with *Prevotella copri* resulted in activation of autoreactive cells in the gut and exacerbated joint inflammation in response to RA autoantigens (17). Pianta et al. reported that CD4^+^ T cells recognizing the autoantigens Filamin A and N-acetylglucosamine-6-sulfatase also recognized similar sequences from *Prevotella*, *Butyricimonas*, and *Parabacteroides* (18). Interestingly, they also identified antibodies against *P. copri* in new-onset RA patients, but not in healthy adults (18). Moreover, *P. copri*-specific IgA responses were strongly associated with serum concentrations of Th1- and Th17-related cytokines. This indicates that the immune response to elevated *P. copri* in the gut may be pivotal in initiating RA. Together, these findings suggest that the microbiota may function as a molecular mimic that triggers autoimmune responses in animal models and RA patients.

## 3 Gut dysbiosis contributes to different clinical features of RA

Chiang et al. used metagenomic analysis to investigate the gut microbiota of RA patients with variable clinical characteristics, and observed that those with positive anti-cyclic citrullinated peptide antibodies had reduced microbial diversity and higher abundance of the *Blautia*, *Akkermansia*, and *Clostridiales* genera (19). A functional link between genes and the arginine deiminase enzyme was also discovered, suggesting a significant role in RA onset (20). This raises the possibility that some gut bacteria may be responsible for protein citrullination and may contribute to the abnormal autoimmunity in RA. The discovery of 21 citrullinated peptides in colon tissues of RA patients suggests that the colon contents create a tolerance site owing to loss of intestinal integrity. The presence of citrullinated antigens may trigger and perpetuate immune responses in RA (21). Patricia et al. investigated the relationship between gut microbiota and inflammatory activities in RA patients (22) and reported that the gut microbiota composition varies with RA disease activities. Despite notable variances across participants, *Collinsella* was strongly associated with cumulative inflammatory burden in RA patients, which is consistent with previous observations (20, 23). Cheng et al. conducted a large-scale cohort study and demonstrated dynamic changes in the gut microbiota and plasma metabolome, as well as their persistent involvement in RA pathogenesis throughout the four distinct RA stages (24). More importantly, they presented solid evidence confirming that microbial invasion of the joint synovial fluid occurs in the fourth stage of RA. Thus, a stage-specific intervention of microbial dysbiosis and metabolic disorders is warranted for improved RA prognosis and prevention.

## 4 Crosstalk between the gut microbiota and different DMARDs

### 4.1 Application of disease-modifying anti-rheumatic drugs in RA

DMARDs are encouraged by international clinical practice guidelines for the therapeutic management of RA (25). These can be administered as a single agent or in combination with other DMARDs, such as methotrexate (MTX), sulfasalazine (SSZ), and leflunomide (LEF). Corticosteroids (Cs) can be either gradually or intermittently administered in conjunction with DMARDs (25). Moreover, chloroquine (CLQ) and hydroxychloroquine (HCQ) are also recommended treatments (26, 27). Treat-to-target treatment (T2T) has recently been proposed as the optimum therapy to achieve clinical remission in RA patients by the rational use of medications. T2T comprises the combined use of MTX + SSZ + HCQ (28). When traditional medications fail to alleviate RA symptoms, clinicians may use biologic DMARDs (bDMARDs) or targeted synthetic DMARDs (tsDMARDs) (1, 29, 30). Currently, conventional synthetic DMARDs (csDMARDs) remain the gold standard for treating RA worldwide.

### 4.2 Gut microbiota regulate the csDMARD metabolic pathway

Despite the recent introduction of various effective therapies, low-dose MTX remains the cornerstone drug for RA treatment and is widely accepted to promote the efficacy of biologics (31, 32). There are three distinct metabolic routes for MTX (33, 34) (**Figure 1**). First, 2,4-diamino-N (10)-methylpteroic acid (DAMPA) is generated as a metabolite of MTX by gut bacteria; carboxypeptidase-G2 (CPDG2) is a bacterial enzyme that forms non-toxic metabolites such DAMPA and glutamate by hydrolyzing MTX (35, 36). *Pseudomonas* species catalyze the production of glutamate through CPDG2 from MTX *in vitro*, suggesting that gut bacteria may govern the availability of active metabolites of drugs as well as control their operational mechanisms (37). Second, the liver’s metabolic pathway is responsible for the biotransformation of MTX to 7-OH-MTX (38). 7-OH-MTX inhibits the dihydrofolate reductase (DHFR) enzyme. DHFR is also present in the gut microbiota, suggesting it may affect the metabolism of drugs, and vice versa, resulting in a reciprocal interaction between the drug and microbial metabolism (34, 38). Third, MTX is converted into polyglutamate within the cells. As it comprises the primary mechanism of immunomodulation, this route is prioritized over others (39, 40). Hence, the main active form of MTX relies on gut homeostasis and intestinal barrier integrity.

Gut microbiota can also indirectly regulate pharmacological metabolism by preserving the integrity of the intestinal barrier. Microbial dysbiosis affects microbial diversity and impacts translocation, immunomodulation, metabolism, and enzymatic degradation in the gut (8, 41). High MTX dosages exert anti-bacterial effects, leading to decreased *Bacteroidetes* and increased *Firmicutes* abundance in the host (42). The abundance of *Bacteroides fragilis*, but not that of *Lactobacillales*, is decreased after MTX administration in mouse models (43, 44). Furthermore, the effects of SSZ on the gut microbiota have also been confirmed despite its use as a monotherapy or in conjunction with MTX (25). Reduction of SSZ into sulfapyridine and 5-aminosalicylic acid (5-ASA/mesalazine) facilitates its metabolism by the gut microbiota through azoreductase-mediated chemical reactions (45). Most 5-ASA is stored in the colon, recirculated via the enterohepatic system, and eliminated in the feces (46). Meanwhile, the anti-inflammatory effects of 5-ASA might be neutralized by microbial arylamine of N-acetyltransferases (NATs) (47). Sulfapyridine can be metabolized in the liver by acetylation by the arylamine NAT-2, hydroxylation, and glucuronidation, all of which contribute to its anti-microbial activity (48–50). Based on the aforementioned factors, the abundance of bacteria belonging to *Bifidobacterium*, *Lactobacillus*, *Enterococcus*, *Clostridium*, *Eubacterium*, and *Bacteroides* genera, which generate azoreductases, as well as those that generate NATs, may have a significant effect on the parameters that define response to RA therapy (51–53).

### 4.3 Regulatory effects of bDMARDs on gut microbiota

bDMARDs are routinely prescribed as an alternative therapy for RA patients who do not respond well to conventional DMARDs. bDMARDs successfully delay RA progression, alleviate symptoms, and improve the overall quality of life by targeting specific proinflammatory cytokines, such as tumor necrosis factor-α (TNF-α), IL-1, and IL-6 (1). As a TNF-α inhibitor, etanercept has a favorable impact on the gut microbiota. Etanercept administration in RA patients was related to a decline in the amount of *Clostridiaceae* and *Deltaproteobacteria* and an increased abundance of *Cyanobacteria* (54). In CIA mice, etanercept treatment decreased diversity and richness of the microbial community; notably, *Escherichia* and *Shigella* became more prevalent, whereas *Clostridium* XIVa, *Tannerella*, and *Lactobacillus* became less common (55). However, our understanding of the impact of biologics on the gut microbiome remains limited. Further research is required to address these knowledge gaps by establishing the mechanisms by which bDMARDs influence the intestinal microbiota diversity in RA.

### 4.4 Regulation of gut microbiota by traditional Chinese medicine

Traditional Chinese medicine (TCM)-based treatments have considerable therapeutic efficacy and cause few side effects in RA (56, 57). Moreover, the potential success of TCM in treating RA may be partially attributed to its ability to alter the composition of the gut microbes (58, 59). Qingluo Tongbi decoction limits inflammatory responses controlled by the gut microbiota and effectively treats arthritis in AIA mice (60). Total paeony glucosides significantly improved microbial taxonomic diversity and increased the relative abundance of some preferable commensal bacteria in CIA rats (61). Xie et al. recently found that the protective effects of ASPS are mediated through the fecal microbiota and inhibited by a concomitant antibiotic cocktail, indicating that gut microbiota may be associated with ASPS (62).

*Tripterygium wilfordii*, a classical Chinese herbal medicine, is commonly used to treat RA in China. It can reduce inflammation and bone damage in RA through various approaches (63). Intestinal microbes, such as *Holdemania fliformis* and *Bacteroides*, are particularly abundant in RA patients; however, the abundance of these bacteria reduced substantially after treatment with glycosides from *T. wilfordii*. Furthermore, the microbiome of RA patients treated with MTX and *T. wilfordii* is abundant in *Prevotella intermedia* compared with those treated with *T. wilfordii* or MTX alone (64). Another independent study revealed that during incubation with Tripterygium glycosides and their active components, the gut microbiota produced various metabolites in the tryptophan (Trp) and phenylalanine (Phe) pathways, including two potentially favorable Trp metabolites: indole propionic acid and indole acetic acid (65). Taken together, these findings suggest that *Tripterygium wilfordii* can impact the host-microbiome composition and modulate metabolite production, underlining their considerable therapeutic potential in RA.

## 5 Impact of gut microbes on the treatment efffcacy of csDMARDs in RA

To date, the pathogenesis of RA remains obscure and conventional therapy either yields inadequate clinical efficacy or has severe adverse events. For example, up to 50% of RA patients who received MTX treatment could not acquire a clinically satisfactory outcome (66, 67); this inadequacy may be attributed to gut microbiota dysbiosis, suggesting that drug metabolism is closely associated with the gut microbiota.

### 5.1 The feedback loop between gut microbiota and csDMARDs

Treatment with DMARDs affects the gut microbiota composition; furthermore, a feedback loop may exist between DMARDs and their effects (8) (**Figure 1**). Significantly, the *Firmicutes* and *Bacteroidetes* phyla could increase owing to RA treatment, which is a desired outcome in this respect (42, 68, 69). Dysbiosis can be caused by either the intrinsic ability to consume a xenobiotic or by extrinsic variables such as drug combination, prescription dosage, or treatment duration. Hence, further investigation into this area is required for the development of optimum RA treatment strategies. Increased abundance of *Clostridium perfringes* in the gut microbiota was observed in an RA patient cohort nonresponsive to standard drug treatment (70). In contrast, the amount of *Clostridium perfringens* was reduced in RA patients who responded well to drug treatment (70, 71). These observations suggest that the microbiota may influence clinical responses to RA treatment. SSZ administration is also associated with decreased *E. coli* and *Bacteroides* abundance, which highlights its anti-bacterial properties (72). Moreover, nonresponse to RA therapy has been linked to MTX consumption, and is currently attributed to increased abundance of *Clostridia* and decreased abundance of *Bacteroidia* (68, 73–75). Certain *Bacteroides* species may be more vulnerable or resistant to csDMARDs, and *Bacteroides* have been demonstrated to possess anti-microbial resistance genes (76, 77).

Variability in DMARDs responses is associated with the presence of bacterial enzymes that catalyze their metabolism. Overexposure to a protein with DHFR activity restores sensitivity to intracellular MTX in strains of medication-resistant *Escherichia coli*. MTX may share compatibility with bacterial DHFR, as in the case of *Escherichia coli* and *Lactobacillus casei* (78–80). Consequently, MTX is deposited in cells harboring mutations in acrA or tolC, which have rendered the efflux pump resistant to multiple AcrAB treatments that rely on tolC inactivity (81). Furthermore, MTX can be converted into MTX polyglutamate by the gut flora (44). Some patients may not respond to the initial strategy of orally administered medicine because the production pathway of tetrahydrofolate reductase, dictated by intestinal metagenomes such as *Bacteroides*, might compete with the DHFR and MTX metabolism in the host, thereby disturbing the anti-inflammatory effects of MTX (68).

### 5.2 Underlying mechanisms of gut microbiota on the treatment efficacy of MTX

In addition to discovering the close association between treatment efficacy and gut microbiota dysbiosis, several studies have comprehensively explored the underlying mechanisms, including immune regulation and metabolic modulation. Herein, we have exemplified the role of MTX, the anchor drug of RA treatment, to illustrate the interactions between gut microbiota and treatment responses. Nayak et al. reported that MTX significantly modifies the human gut microbiome. Despite differing drug susceptibility between strains, the action mechanism against DHFR is seemingly conserved in human and bacterial cells (42). The gut microbiota of RA patients responded differently to MTX treatment in terms of alterations in bacterial taxa and abundance in gene families. Immune activation was suppressed after transplanting post-treatment samples into germ-free mice exposed to inflammatory triggers, permitting the detection of MTX-modulated bacterial taxa associated with intestinal and splenic immune cells (42). Artacho et al. revealed a significant correlation between the abundance of gut bacterial taxa and their genes, particularly orthologs involved in methotrexate and purine metabolism, with clinical responses (82). Additionally, they created a microbiome-based model that predicts MTX non-response in a different set of patients. Intriguingly, clinical response was strongly associated with MTX levels remaining after *ex vivo* incubation with distal gut samples from pre-treatment RA patients, implying a direct impact of the gut microbiota on MTX metabolism and therapeutic efficacy. More recently, using machine learning Han et al. discovered that the composition of genes involved in MTX metabolism differed significantly between the response and non-response groups (83). These genes were predominantly related to *Firmicutes* and *Bacteroidetes*. Furthermore, they demonstrated that the catabolic ability of the drug in the gut microbiota is closely associated with the response mechanism to MTX in RA patients, and proposed that metabolic capability is a critical component in determining the host response to MTX.

## 6 Conclusion and perspectives

The gut microbiota has been extensively studied over the last decades, and its importance in health and disease states has been established. Gut microbiota influence almost every biological process within the host, and microbiota dysbiosis is associated with compromised immunological tolerance and RA development. Moreover, alterations in the gut microbiota have been linked to RA disease activity, even before clinical arthritis onset. Analyzing the gut microbiota has provided novel insights into variables that promote or limit the sensitivity to disease, and has become a feasible method for predicting and reducing RA occurrence. The human gut microbiota and its enzymatic products can also directly and/or indirectly influence drug bioavailability, clinical efficacy, and toxicity. Conversely, certain medications and active ingredients can influence the immune system by modifying the gut microbiota composition, thus strengthening the host defenses. Despite significant abnormalities in specific microbial communities being associated with RA progression, several critical aspects must be addressed to facilitate development of gut microbiota-targeted treatments. First, future studies need to establish the causes of dysbiosis and to determine exactly how and when gut dysbiosis influences RA development. Second, the association of the disease with altered microbial composition and the mechanistic pathways influencing RA development must be elucidated, to acquire effective diagnostic, prognostic, and therapeutic targets. Third, it is necessary to understand the mechanism of the effects DMARDs exert on the gut microbiota and the implications of gut dysbiosis on the modulation of treatment response, in order to optimize therapeutic strategies that restore microbial symbiosis in RA patients. Collectively, further research into these unresolved topics would help promote treatment efficacy, reduce toxicity risk, and improve RA clinical outcomes.

## Author contributions

JF conceived and wrote the manuscript. TJ collected the references. DH supervised the study and revised the manuscript. All authors contributed to the article and approved the submitted version.
